# Divergent Spatial Learning, Enhanced Neuronal Transcription, and Blood–Brain Barrier Disruption Develop During Recovery from Post-Injury Sleep Fragmentation

**DOI:** 10.1089/neur.2023.0018

**Published:** 2023-09-21

**Authors:** Zoe M. Tapp, Cindy Ren, Kelsey Palmer, Julia Kumar, Ravitej R. Atluri, Julie Fitzgerald, John Velasquez, Jonathan Godbout, John Sheridan, Olga N. Kokiko-Cochran

**Affiliations:** ^1^ Department of Neuroscience, College of Medicine, College of Dentistry, The Ohio State University, Columbus, Ohio, USA.; ^2^ Chronic Brain Injury Program, College of Dentistry, The Ohio State University, Columbus, Ohio, USA.; ^3^ Institute for Behavioral Medicine Research, Neurological Institute, College of Dentistry, The Ohio State University, Columbus, Ohio, USA.; ^4^ Division of Biosciences, College of Dentistry, The Ohio State University, Columbus, Ohio, USA.

**Keywords:** Barnes maze, blood–brain barrier, lateral fluid percussion, sleep fragmentation

## Abstract

Traumatic brain injury (TBI) causes pathophysiology that may significantly decrease quality of life over time. A major propagator of this response is chronic, maladaptive neuroinflammation, which can be exacerbated by stressors such as sleep fragmentation (SF). This study determined whether post-TBI SF had lasting behavioral and inflammatory effects even with a period of recovery. To test this, male and female mice received a moderate lateral fluid percussion TBI or sham surgery. Half the mice were left undisturbed, and half were exposed to daily SF for 30 days. All mice were then undisturbed between 30 and 60 days post-injury (DPI), allowing mice to recover from SF (SF-R). SF-R did not impair global Barnes maze performance. Nonetheless, TBI SF-R mice displayed retrogression in latency to reach the goal box within testing days. These nuanced behavioral changes in TBI SF-R mice were associated with enhanced expression of neuronal processing/signaling genes and indicators of blood–brain barrier (BBB) dysfunction. Aquaporin-4 (AQP4) expression, a marker of BBB integrity, was differentially altered by TBI and TBI SF-R. For example, TBI enhanced cortical AQP4 whereas TBI SF-R mice had the lowest cortical expression of perivascular AQP4, dysregulated AQP4 polarization, and the highest number of CD45^+^ cells in the ipsilateral cortex. Altogether, post-TBI SF caused lasting, divergent behavioral responses associated with enhanced expression of neuronal transcription and BBB disruption even after a period of recovery from SF. Understanding lasting impacts from post-TBI stressors can better inform both acute and chronic post-injury care to improve long-term outcome post-TBI.

## Introduction

Traumatic brain injury (TBI) is the leading cause of long-term injury-induced disability with TBI survivors experiencing pain, cognitive deficits, and psychiatric disorders post-injury.^1^ Chronic, maladaptive neuroinflammation has been a focus of both pre-clinical and clinical neurotrauma research as a major propagator of long-term impairment.^2,3^ Previous work shows that chronic reactivity of the resident brain macrophage, microglia, is associated with lasting cognitive deficits in TBI survivors.^4^ This is recapitulated in experimental models of TBI given that depletion or turnover of microglia after injury attenuates neuroinflammation, improves neuronal function, and rescues behavioral deficits.^5–8^

Stress enhances neuroinflammation and promotes behavioral complications, including anxiety and depression. For example, pre-clinical psychosocial stress enhances anxiety-like behavior attributable to microglial-dependent recruitment of proinflammatory monocytes to the brain.^9–13^ Restraint stress in mice also increases neuroinflammatory markers associated with increased anxiety- and depressive-like behavior and cognitive deficits.^14–16^ There is mounting pre-clinical and clinical evidence that TBI causes dysfunctional stress responses through suppressed reactivity of the hypothalamic-pituitary-adrenal axis. Aberrant stress responses post-TBI can thus exacerbate injury-induced inflammation, contributing to compromised recovery and long-term deficits.^17^ Clinical TBI populations are less resilient to challenges that cause stress after their injury^18^ and are thus more susceptible to stress-induced exacerbations such as psychiatric disorders and cognitive impairment.^19^

Many modalities of stress result in sleep disturbances; thus, sleep disruption provides a common biological pathway through which to study the effects of stress.^20–23^ Sleep disturbances are a particularly relevant stressor post-TBI, given that TBI-induced sleep disturbances are prevalent chronically after injury,^24–26^ and injured populations are susceptible to environmental sleep disruption because of environments like combat theaters or hospitalization.^27,28^ Further, the combination of low resilience and poor sleep after TBI significantly contributes to poor neurological outcome long-term after injury.^29,30^

Stress compromises post-injury recovery; however, TBI also impedes recovery from stress.^31^ This delayed resolution of stress could thus create a feed-forward cycle of dysfunctional stress response and post-injury consequences. Pre-clinical psychosocial stress has lasting effects by sensitizing neuroinflammatory and behavioral responses to subsequent stressors.^32–34^ Unpredictable and restraint stress in mice also causes lasting neuroinflammatory and behavioral deficits once the stressor has concluded, particularly in hippocampal-mediated spatial learning and memory.^35–37^ Sleep disturbances similarly have long-lasting consequences, such as lasting spatial learning and memory deficits, in pre-clinical studies.^38^ These lasting effects of stress could synergize and be exacerbated with previous TBI experience. Indeed, acute sleep disruption post-TBI causes enhanced neuroinflammation, even with a subsequent acute period of undisturbed sleep.^39^

We have shown that 30 days, defined as chronic,^40–42^ of sleep fragmentation (SF) after lateral fluid percussion injury (lFPI) causes enhanced microglial inflammation and compromises hippocampal-mediated cognition.^43^ The present study expands upon these studies and determines whether a period of recovery alleviates neuroinflammatory and behavioral consequences of post-TBI SF relative to TBI alone. We thus induced 30 days of post-TBI SF followed by a period of undisturbed sleep, or recovery, for an additional 30 days. Hippocampal-mediated behavior was determined with the Barnes maze task (BMT), and inflammatory outcomes were determined by gliosis and transcriptome analysis. We found that post-TBI SF causes persistent behavioral and inflammatory consequences compared to TBI alone given that there were persistent within-day spatial learning deficits, enhanced neuronal transcription, and blood–brain barrier (BBB) deficits with post-TBI SF even with a period of recovery.

## Methods

### Subjects

Equal numbers of 8- to 10-week-old male and female C57BL/6 mice were obtained from Charles River Laboratories (Wilmington, MA). Mice were group-housed by sex and maintained at 23°C under a 12h:12h light/dark cycle with lights on at 7:00 am. For all experiments, mice were euthanized by CO_2_ asphyxiation in constant flow rates and environment to minimize animal distress. Conditions were in accordance with the National Institutes of Health Guidelines for the Care and Use of Laboratory Animals and approved by the Institutional Animal Care and Use Committee of The Ohio State University.

### Experimental study design

The objective of this study was to determine whether a period of recovery is sufficient to alleviate the behavioral and inflammatory effects of post-injury SF. Each cohort received either sham or lFPI and were then left undisturbed (control; CON) or exposed to daily, transient SF for 30 days post-injury (DPI). All mice remained undisturbed 30–60 DPI, allowing recovery in mice exposed to post-injury SF (termed SF-R) and resulting in a 2 (sham, TBI) × 2 (CON, SF-R) factorial design. Replicate numbers were based on previous publications using α = 0.05.^44–46^ The BMT was conducted with four cohorts (total, *n* = 12 per group). Two of these cohorts were used for immunofluorescent analyses (*n* = 6 per group). Gene expression analyses were conducted with the other two cohorts (*n* = 6 per group: sham CON, TBI CON, and TBI SF-R; *n* = 5 per group: sham SF-R). A blinded investigator completed all subsequent data analysis.

### Lateral fluid percussion injury

The lFPI was performed as previously described.^43^ Briefly, after a midline incision, a 3-mm craniectomy was trephined over the right parietal bone, leaving the dura mater intact, and a modified Leur-Loc needle hub was affixed over the craniectomy with cyanoacrylate. After 24 h to allow recovery from the craniectomy procedure, mice were anesthetized for 4 min with 4% isoflurane and immediately connected to the fluid percussion injury device. A pendulum delivered a fluid pulse onto the exposed dura mater for mice designated to receive TBI. After the fluid pulse, the hub was removed and the incision was closed with two 7-mm surgical clips. Sham mice received the same amount of isoflurane and post-hub removal care, but no fluid pulse injury. Righting reflex was assessed as a measure of injury severity to ensure consistency of sham or injured status.

### Sleep fragmentation and recovery

SF was performed using SF chambers (model 80391; Lafayette Instrument Company Inc, Lafayette, IN), as previously described.^43^ Briefly, a sweeper bar moved across the bottom of the home cage every 2 min for 4 h after the onset of the light cycle (7:00 am to 11:00 am). Mice were group-housed by sex and had *ad libitum* access to food and water. CON mice were housed in control housing (model 223581; Allentown, LLC, Allentown, NJ) and also group-housed by sex with *ad libitum* access to food and water. After 30 days, mice receiving SF were taken out of the SF chambers and allowed to recover in control housing.

### Barnes maze

BMT testing occurred from 45 to 49 DPI with a probe trial at 52 DPI. For three trials each day, mice were placed in the middle of the maze and allowed to explore for 2 min. Each trial ended when the mouse entered and remained in the escape box for 3 sec or after 2 min had fully elapsed, after which mice were placed in the escape box for 30 sec. The maze and escape box were cleaned with 70% ethanol between each trial. Acquisition index was defined as the mean difference between trials 1 and 3 within each testing day. Savings index was defined as the mean difference between trial 3 of one testing day and trial 1 of the following testing day. Seventy-two hours after the last testing day, mice freely explored the maze with no escape box for 30 sec for a single probe trial.

### Immunofluorescent labeling

Immunofluorescent labelling was performed as previously described.^43^ Briefly, mice were transcardially perfused with phosphate-buffered saline and 4% paraformaldehyde. After post-fixation and cryoprotection, brains were sectioned at 30 μm on a LeicaCM1800 cryostat (Leica Biosystems, Wetzlar, Germany). Tissue was incubated with primary antibody overnight at 4°C: rabbit anti-mouse ionized calcium-binding adaptor molecule 1 (Iba-1; 1:1000; 019-19741; Wako Chemicals USA, Richmond, VA); goat anti-mouse glial fibrillary acidic protein (GFAP; 1:1000; NC0421235; Invitrogen, Carlsbad, CA); rat anti-mouse CD45 (1:1000; MCA1388; Bio-Rad Laboratories, Hercules, CA); and rabbit anti-mouse aquaporin-4 (AQP4; 1:2000; HPA014784; Sigma-Aldrich, St. Louis, MO). Tissue was then washed and incubated with secondary antibody for 1 h at room temperature: donkey anti-rabbit Alexa Fluor^®^ 647 (1:500; A31573; Life Technologies, Thermo Fisher Scientific, Waltham, MA); donkey anti-rabbit Alexa Fluor 594 (1:500; A21207; Life Technologies, Thermo Fisher Scientific); donkey anti-goat Alexa Fluor 647 (1:500; A32849; Life Technologies, Thermo Fisher Scientific); and donkey anti-rat Alexa Fluor 594 (1:500; A32744; Life Technologies, Thermo Fisher Scientific). Sections were then mounted and cover-slipped with Fluoromount-G (Invitrogen).

For Iba-1 and GFAP, slides were imaged with an EVOS FL Auto2 Imaging System (Thermo Fisher Scientific) at 20 × magnification with two to three images per region of interest, and percent area was quantified with ImageJ Software (NIH, Bethesda, MD). For CD45, slides were imaged with the EVOS Imaging System at 10 × magnification with non-overlapping images of the medial cortex, lesion area, and lateral cortex to span the entire ipsilateral cortex. Two to three images per region of interest were taken for each mouse, and the numbers of CD45^+^ cells >5 μm from edge of the tissue were manually counted to ensure the quantification of only infiltrating cells. AQP4 was imaged on an SP8 Leica upright confocal microscope in optical section Z-stacks of 8–10 images at 20 × magnification using Leica Application Suite X Imaging software (Leica Biosystems). AQP4 intensity and polarization were determined using custom in-house software as previously described.^39,47^ AQP4 polarization was calculated as the ratio of perivascular to parenchymal (20 μm from vessel) AQP4 immunofluorescence. Representative images for AQP4 were cropped from the original analysis image to emphasize the large vessel used in analysis. Representative images for CD45 had picture correction for brightness (40%) and contrast (−20) to highlight positive labeling.

### NanoString nCounter analysis

Gene expression analyses were performed as previously described for NanoString nCounter analysis.^43^ After euthanasia at 60 DPI, microdissected ipsilateral cortex was snap-frozen in liquid nitrogen (−196°C) and whole cortical RNA was isolated using the Tri-Reagent protocol (Sigma-Aldrich). RNA quality and concentration were determined by the Agilent 2200 TapeStation assay (Agilent Technologies, Santa Clara, CA), and analysis was performed by the OSU Comprehensive Cancer Center Genomics Shared Resource Facility using the Glial Signaling Panel (NanoString Technologies, Seattle, WA).

### Statistical analysis

Statistical analysis for righting time, BMT, and immunofluorescence was performed with Prism software (version 9.0.0; GraphPad Software Inc., La Jolla, CA). Sex differences were considered for interaction effects by three-way analysis of variance (ANOVA) in righting times, BMT learning indices, and immunofluorescent labeling and were observed in cortical Iba-1 labeling and perivascular AQP4 expression. No sex differences were observed in all other analyses, so males and females were pooled. For righting time, BMT learning indices and probe trial, and immunofluorescence, a two-way ANOVA was performed with injury (sham, TBI) and condition (CON, SF-R) as independent variables.

Main effects were followed with Bonferroni's correction for multiple comparisons. Interaction effects were followed with Tukey's correction for multiple comparisons. For BMT duration and AQP4 analysis along the vessel, a three-way ANOVA with repeated measures was performed with injury (sham, TBI) and condition (CON, SF-R) as independent variables and testing day for BMT or distance along the vessel for AQP4 as repeated measures. Geisser-Greenhouse's epsilon correction was determined and reported, when necessary. Significant main and interaction effects are reported. For NanoString analysis, a two-way ANOVA was performed on normalized gene expression copy numbers using SPSS statistical software (SPSS, Inc., Chicago, IL) with injury (sham, TBI) and condition (CON, SF-R) as independent variables. Significant main or interaction effects were then followed by group-wise comparisons from DESeq2 (R software; The R Foundation for Statistical Computing, Vienna, Austria). Statistical significance was determined as *p* < 0.05 for all data and is presented as mean ± standard error of the mean (SEM).

## Results

### Traumatic brain injury sleep fragmentation with recovery alters spatial learning acquisition in the Barnes maze task

**FIG. 1.**
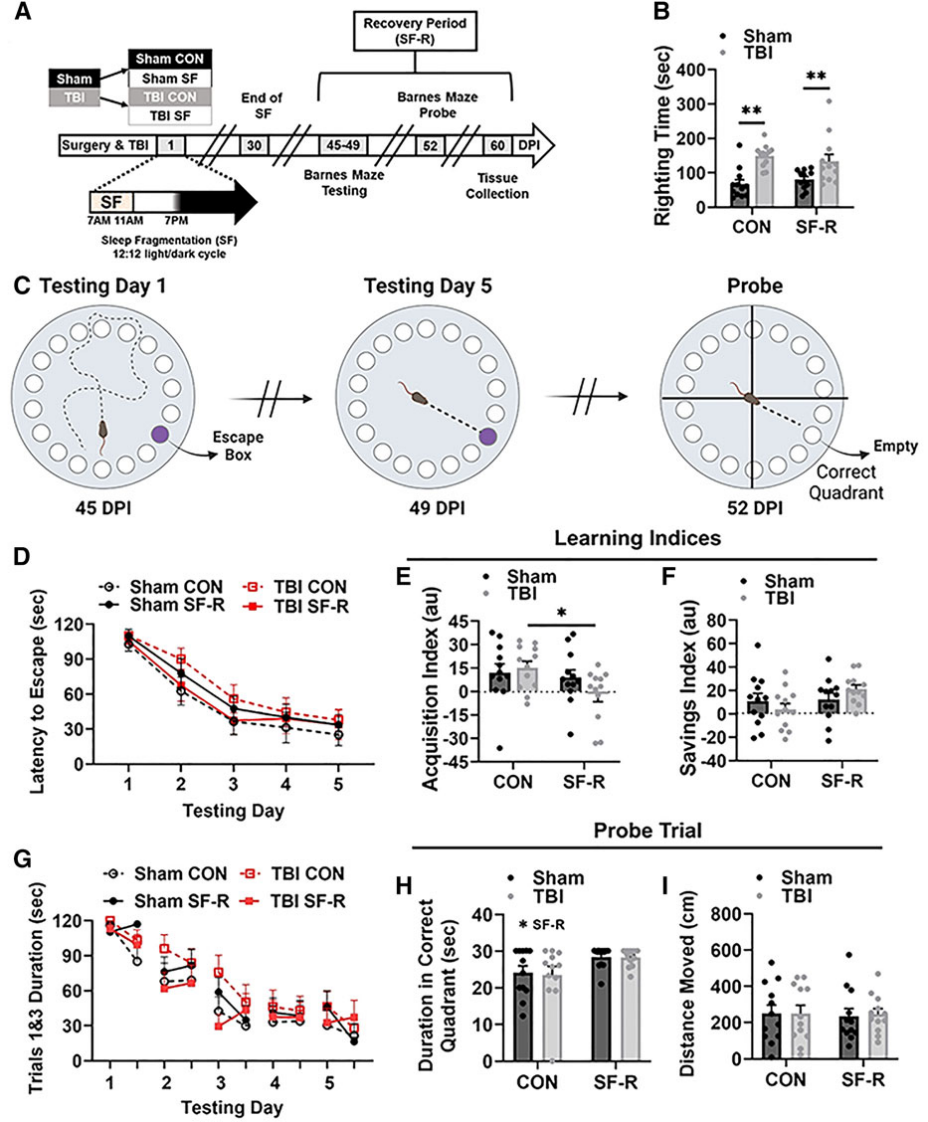
TBI SF-R alters spatial learning acquisition in the Barnes maze task. (**A**) Equal numbers of 8- to 10-week-old mice received sham or TBI injury and were assigned to remain undisturbed (CON) or receive SF for 30 days. Timeline inset shows specific timing of the daily, transient exposure to 4 h of SF beginning at the onset of the light period. This same SF protocol was given daily for a full 30 DPI. At 30 DPI, all mice were left undisturbed for an additional 30 days, to 60 DPI. (**B**) An *a priori* analysis shows that TBI increased righting time compared to sham (main effect TBI, *p* < 0.01; Bonferroni multiple comparisons, *p* < 0.05 between sham and TBI groups), with no differences between groups preceding SF-R. (**C**) From 45 to 49 DPI, mice underwent training for the Barnes maze task and then a 30-sec probe trial testing at 52 DPI. (**D**) All groups learned to escape the maze over the 5 days of testing, with no differences in escape latency between groups. (**E**) TBI SF-R decreased the acquisition index compared to TBI CON, indicating within-day learning deficits (main effect SF-R, *p* = 0.054; Bonferroni multiple comparisons, *p* < 0.05). (**F**) TBI SF-R had the highest mean savings index, but there were no significant differences between groups. (**G**) Acquisition and savings indices are demonstrated by plotting trials 1 and 3 for each testing day. (**H**) Mice exposed to SF-R spent more time in the correct quadrant during the probe trial at 52 DPI (main effect SF-R, *p* < 0.01); this was not associated with any differences in distance traveled (**I**). *N* = 48; *n* = 12 per group. Data presented as mean ± SEM. *p < 0.05, **p < 0.01. CON, control; DPI, days post-injury; SEM, standard error of the mean; SF, sleep fragmentation; SF-R, sleep fragmentation with recovery; TBI, traumatic brain injury.

This study determined whether a period of recovery would be sufficient to alleviate the behavioral and inflammatory effects of post-injury SF (Fig. 1A). Righting times were taken as a measure of injury severity immediately post-TBI and *a priori* to SF (Fig. 1B). Consistent with previous reports,^39,43^ TBI increased righting time (main effect TBI; *F*_(1, 44)_ = 25.12, *p* < 0.01), indicating that any subsequent differences between sham or TBI groups were attributable to SF-R.

The BMT assessed cognitive function 45–49 DPI and with a probe trial at 52 DPI (Fig. 1C). Latency to escape decreased across testing days in all experimental groups (main effect testing day; Geisser-Greenhouse's epsilon = 0.6903; *F*_(2.761, 121.5)_ = 66.88, *p* < 0.01; Fig. 1D); however, no between-group differences were detected. Two learning indices demonstrated within- and between-day performance where positive values represented learning.^48^ TBI SF-R mice had the lowest acquisition index, representing decreased within-day learning, contributing to a main effect of SF-R (*F*_(1, 44)_ = 4.222, *p* < 0.05; Fig. 1E). *Post hoc* analysis showed that the acquisition index was significantly lower with TBI SF-R compared to TBI CON. Although TBI SF-R mice showed the highest savings index, representing between-day learning, no significant between-group differences were detected (Fig. 1F). Together, these learning indices show that TBI causes unique within- and between-day learning profiles subsequent to a period of recovery after SF.

To visualize the time-dependent changes of these indices, trial 1 and 3 latencies were plotted across each testing day (Fig. 1G). During the probe trial, SF-R increased time spent in the correct quadrant regardless of TBI (main effect SF-R; *F*_(1, 44)_ = 7.927, *p* < 0.01; Fig. 1H). *Post hoc* analysis found no significant between-group differences, but this increased duration in the correct quadrant was not attributable to changes in distance traveled (Fig. 1I) and is consistent with SF-R mice having the highest mean savings index during testing days.

### Traumatic brain injury causes lasting microglial and astrocytic reactivity lateral of the lesion area and in the ipsilateral CA1

**FIG. 2.**
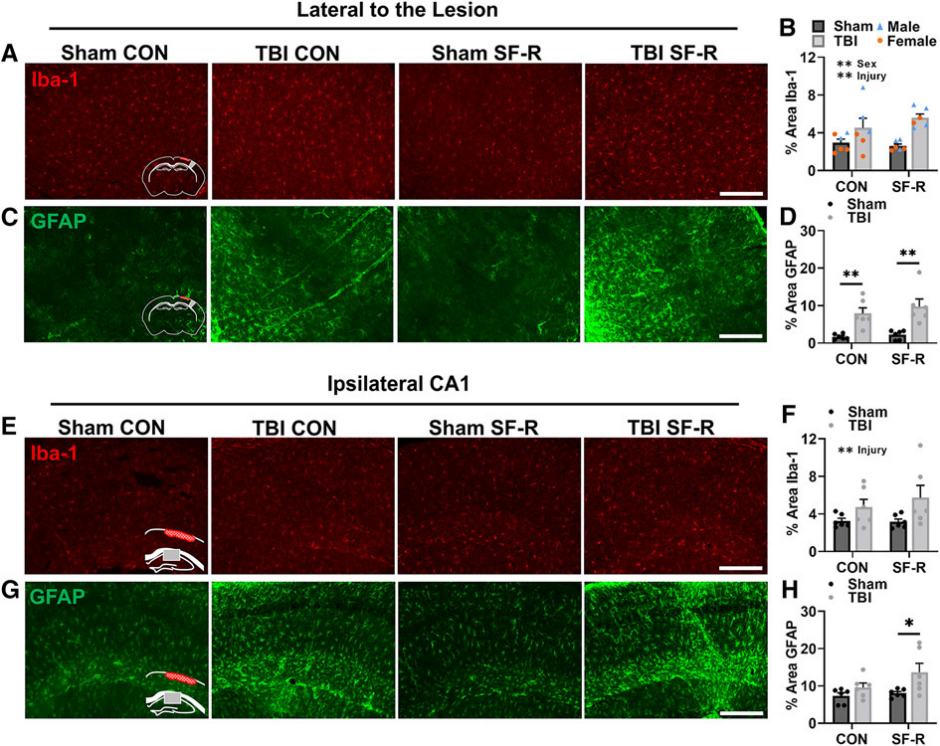
TBI causes lasting astrocytic reactivity lateral of the lesion area and in the ipsilateral CA1. Representative images of Iba-1 (**A**) and GFAP (**C**) lateral to the lesion area, equivalent to the somatosensory cortex. (**B**) TBI increased percent area of Iba-1 (main effect TBI, *p* < 0.01) with an influence of sex (main effect sex, *p* < 0.01). Orange circles indicate females, and blue triangles indicate males. (**D**) TBI also increased percent area of GFAP in the somatosensory cortex (main effect TBI, *p* < 0.01; Bonferroni multiple comparisons, *p* < 0.01 between sham and TBI groups). These increases were independent of SF-R. Representative images of Iba-1 (**E**) and GFAP (G) in the ipsilateral CA1. (**F**) TBI increased percent area of Iba-1 (main effect TBI, *p* < 0.01), with no influence of SF-R. (**G**) TBI SF-R had the highest percent area GFAP in the ipsilateral CA1 compared to sham SF-R (main effect TBI, *p* < 0.05; Bonferroni multiple comparisons, *p* < 0.05). *N* = 24; *n* = 6 per group. Data presented as mean ± SEM. Scale bar = 200 μm. *p < 0.05, **p < 0.01. CON, control; GFAP, glial fibrillary acidic protein; Iba-1, ionized calcium-binding adaptor molecule 1; SEM, standard error of the mean; SF-R, sleep fragmentation with recovery; TBI, traumatic brain injury.

The somatosensory cortex is vital for spatial tasks because of the necessity of the barrel cortex for environmental navigation.^49^ The somatosensory cortex also has hippocampal afferents to enhance hippocampal-dependent spatial tasks.^50^ We thus determined microglial and astrocytic reactivity in these regions of interest. We first determined microglial morphological restructuring, as an indicator of reactivity, lateral to the lesion by the Iba-1 percent area (Fig. 2A,B). There was a main effect of sex (*F*_(1, 16)_ = 10.88, *p* < 0.01) given how *post hoc* analysis showed that male mice had a higher Iba-1 percent area than female mice with TBI alone. Overall, TBI increased microglial reactivity lateral to the lesion area (main effect TBI; *F*_(1, 16)_ = 19.08, *p* < 0.01), with *post hoc* analysis showing a significant increase in Iba-1 percent area with male TBI SF-R compared to male sham SF-R.

We next determined astrocytic reactivity by GFAP percent area (Fig. 2C,D). TBI increased astrocytic reactivity lateral to the lesion (main effect TBI; *F*_(1, 20)_ = 30.03, *p* < 0.01) regardless of SF-R given how *post hoc* analysis found that TBI increased GFAP labeling compared to sham in CON and SF-R mice. TBI also increased microglial reactivity in the ipsilateral CA1 (main effect TBI; *F*_(1, 20)_ = 6.217, *p* < 0.05), with no significant *post hoc* comparisons (Fig. 2E,F). Notably, there was a main effect of TBI with astrocytic reactivity in the ipsilateral CA1 (*F*_(1, 20)_ = 7.694, *p* < 0.05), and *post hoc* analysis showed that this effect was driven by TBI SF-R compared to sham SF-R (Fig. 2G,H). Astrocytes play a major role in the pathogenesis of TBI^51^; thus, this persistent injury-induced reactivity within the hippocampal CA1 only with SF-R could represent an area of interest for mediating chronic outcome after injury.

### Traumatic brain injury sleep fragmentation with recovery enhances transcription associated with neuronal signaling and blood–brain barrier disruption

**FIG. 3.**
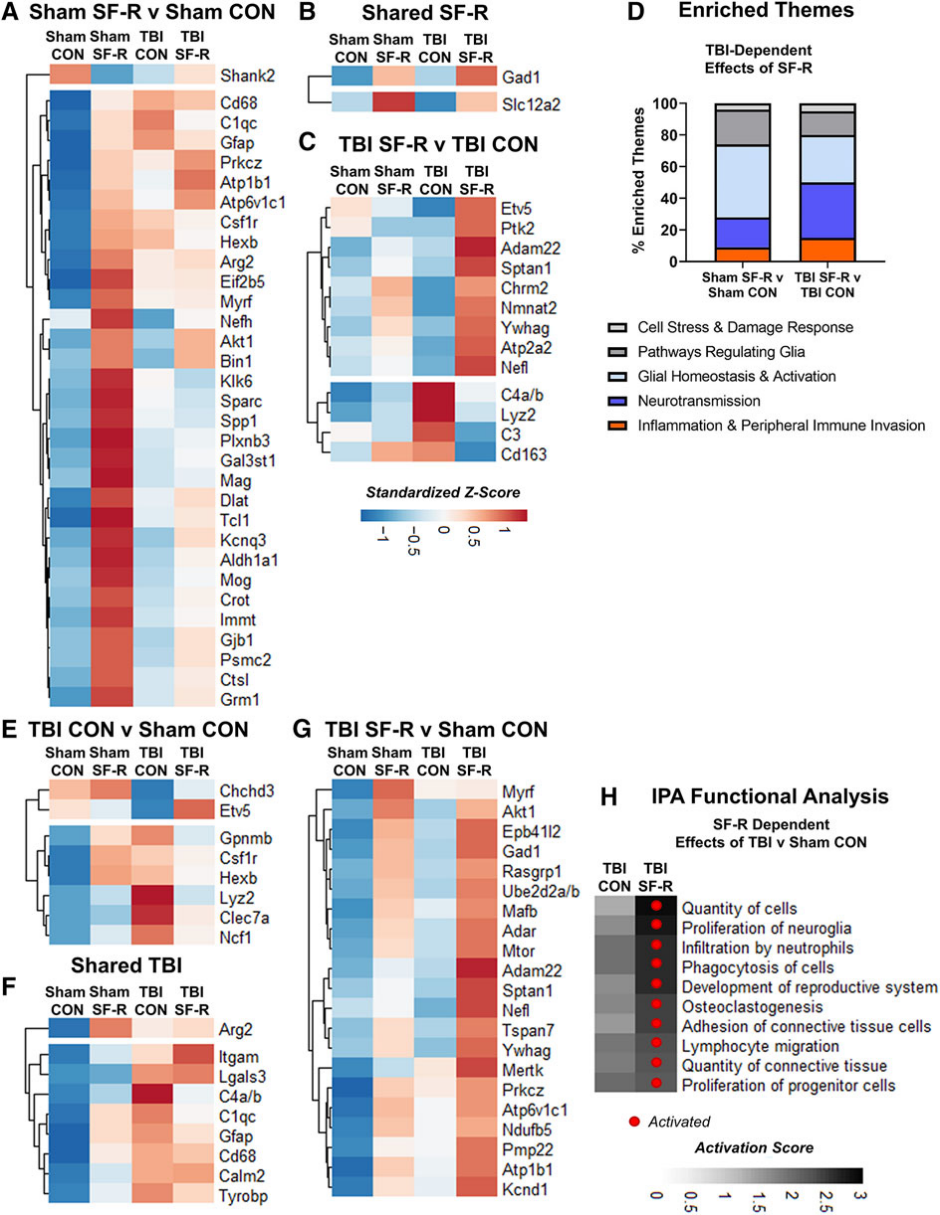
TBI SF-R enhances transcription associated with neuronal signaling and BBB disruption. Heatmaps showing standardized Z-score of DEGs (**A**) between sham SF-R and sham CON, (**B**) increased by SF-R compared to CON regardless of TBI, and (**C**) between TBI SF-R and TBI CON. (**D**) Injury-dependent SF-R effects on enriched themes, as defined by nanoString. TBI SF-R versus TBI CON has a larger percent of “Neurotransmission” and “Inflammation & Peripheral Immune Invasion” associated genes compared to Sham SF-R versus sham CON. Heatmaps showing standardized Z-score of DEGs (**E**) between TBI CON and sham CON, (**F**) increased by TBI regardless of SF-R, (**G**) between TBI SF-R versus sham CON. (**H**) IPA functional analysis of top activated pathways in TBI CON and TBI SF-R compared to sham CON. *N* = 23; *n* = 6 per group sham CON, TBI CON, and TBI SF-R; *n* = 5 per group sham SF-R. BBB, blood–brain barrier; CON, control; DEGs, differentially expressed genes; IPA, Ingenuity Pathway Analysis; SF-R, sleep fragmentation with recovery; TBI, traumatic brain injury.

We next determined the transcriptomic signature of TBI and SF-R with NanoString nCounter mRNA analysis in the ipsilateral cortex. There was a robust SF-R effect alone with 32 differentially expressed genes (DEGs) between sham SF-R and sham CON (Fig. 3A). Many of these genes are associated with glial homeostasis (*Aldh1a1*, *Spp1*, and *Csf1r*) and myelogenesis (*Gal3st1*, *Mag*, and *Mog*; Fig. 3A). SF-R influences inhibitory neuron regulation (Fig. 3B) given that *Gad1* contributes to gamma-aminobutyric acid (GABA) synthesis^52^ and *Slc12a2* encodes NKCC1, which regulates GABAergic signaling^53^ (Fig. 3B). This neuronal signature was further evident in the 13 DEGs of TBI SF-R compared to TBI CON (Fig. 3C). Genes associated with neuronal signaling/maintenance (*Ywhag*, *Chrm2*, and *Sptan1*) were increased with TBI SF-R, indicating enhanced synaptogenesis or neurotransmission. Genes associated with cellular metabolism and responses to oxidative stress (*Etv5*, *Nmnat2*, and *Atp2a2*) were also increased with TBI SF-R, indicating mitochondrial dysfunction, which is evident with TBI and stress and contributes to chronic neuroinflammation.^54^

Genes decreased by TBI SF-R compared to TBI CON (*C3*, *C4a/b*, *Cd163*, and *Lyz2*) were all associated with macrophage reactivity. BBB disruption genes were also altered by TBI SF-R compared to TBI CON. For example, *Cd163* is a marker of perivascular macrophages that helps to maintain the BBB, and *Ptk2* indicates metalloproteinase activity that degrades the BBB.^55^ This altered neuronal signaling and dysfunctional BBB signature was further demonstrated through enriched themes by NanoString (Fig. 3D). Sham SF-R versus sham CON had the most enrichment in glial homeostasis themes whereas TBI SF-R versus TBI CON had an enrichment of neurotransmission and infiltration of peripheral immune cells.

We next determined unique injury effects by comparing TBI SF-R and TBI CON each to sham CON. TBI CON uniquely increased the genes associated with microglial/macrophage-mediated inflammation (*Hexb*, *Csf1r*, and *Gpnmb*) and mitochondrial/oxidative stress (*Lyz2*, *Ncf1*, *Chchd3*, and *Etv5*; Fig. 3E). Many genes were increased in both TBI groups compared to sham CON and were the most highly expressed in TBI CON (Fig. 3F). In the unique TBI SF-R injury response (Fig. 3G), *Nefl*, which was significantly increased with TBI SF-R compared to all groups, encodes the neurofilament-light chain protein, a promising biomarker for TBI experience and outcome in both pre-clinical and clinical research.^56^ Genes associated with neuronal maintenance and excitability (*Sptan1*, *Kcnd1*, *Ywhag*, *Adar*, and *Epb41l2*) were highest with TBI SF-R, mirroring the unique injury-dependent SF-R neuronal signature.^57^ Genes related to hematopoiesis and lymphocyte homeostasis/differentiation (*Mafb*, *Rasgrp1*, *Akt1*, and *mTor*) are also increased uniquely with TBI SF-R. The Ingenuity Pathway Analysis (IPA) functional analysis revealed that several pathways activated only with TBI SF-R related to peripheral immune cell differentiation, migration, and proliferation (Fig. 3H). In conjunction with injury-dependent SF-R-enriched themes, the unique genetic landscape of TBI SF-R indicates long-lasting changes in neuronal maintenance, BBB damage, and infiltration of immune cells.

### Traumatic brain injury sleep fragmentation with recovery disrupts aquaporin-4 expression associated with presence of infiltrating cells

**FIG. 4.**
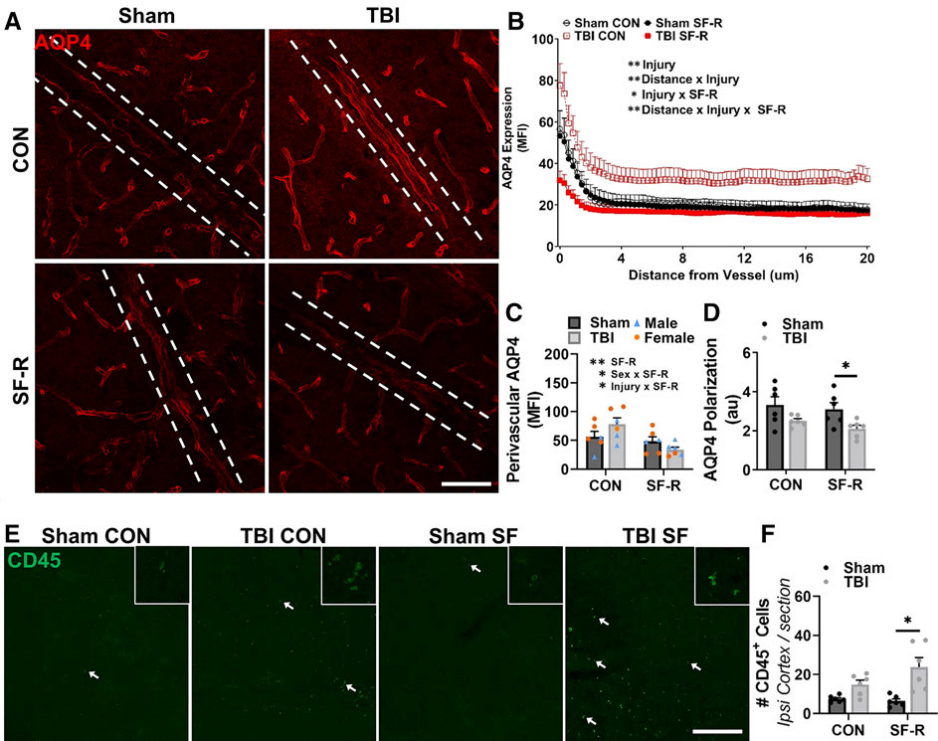
TBI SF-R disrupts AQP4 expression associated with the presence of infiltrating cells. (**A**) Representative images of AQP4 labeling of large vessels (>15 μm) in the ipsilateral cortex. (**B**) TBI CON and TBI SF-R differentially influence AQP4 expression along the vessel from the perivascular wall to 20 μm into the parenchyma. (**C**) TBI SF-R has the lowest perivascular AQP4 expression, in part driven by an interaction sex effect. (**D**) AQP4 polarization, showing the proportion of perivascular expression to parenchymal expression at 20 μm, is decreased by TBI SF-R to sham SF-R, indicating BBB disruption (main effect TBI, *p* < 0.04; Bonferroni multiple comparisons, *p* < 0.05). (**E**) Representative images of CD45 labeling in the ipsilateral cortex with inset of CD45^+^ cells. (**F**) TBI SF-R has the highest presence of CD45^+^ cells in the ipsilateral cortex (main effect TBI, *p* < 0.05; Bonferroni multiple comparisons, *p* < 0.05). *N* = 22; *n* = 6 per group. Data presented as mean ± SEM. Scale bar = 50 μm. White arrows indicate examples of positive labeling. *p < 0.05, **p < 0.01. AQP4, aquaporin-4; BBB, blood–brain barrier; CON, control; SEM, standard error of the mean; SF-R, sleep fragmentation with recovery; TBI, traumatic brain injury.

Because of the BBB damage genetic signature, we next measured AQP4 polarization as a marker of BBB damage. AQP4 is selectively expressed on the end-feet of astrocytes and helps to maintain the osmotic balance of the BBB.^58,59^ In response to a challenge, such as TBI or stress, astrocytes may become reactive and the localization of AQP4 becomes dysregulated and compromises the BBB.^60^ We thus labeled for AQP4 as a marker of BBB disruption (Fig. 4A).

AQP4 labeling was highest at the perivascular wall and lowest in the parenchymal space in all groups (main effect distance; *F*_(1.593, 31.86)_ = 74.12, *p* < 0.01; Fig. 4B). TBI CON had the highest expression of AQP4 at the perivascular wall and in the parenchyma (main effect TBI, *F*_(1, 20)_ = 5.276, *p* < 0.05; interaction distance × injury, *F*_(70, 1400)_ = 4.413, *p* < 0.01). This response was altered by SF-R, given that TBI SF-R had the lowest perivascular expression and parenchymal expression was similar to sham groups (interaction injury × SF-R; *F*_(1, 20)_ = 4.748, *p* < 0.05). Specifically at the perivascular wall, TBI SF-R had the lowest AQP4 expression (main effect SF-R; *F*_(1, 16)_ = 9.395, *p* < 0.01; Fig. 4C). Female TBI CON animals had the highest expression of perivascular AQP4 (interaction sex × SF-R; *F*_(1, 16)_ = 5.247, *p* < 0.05).

*Post hoc* analysis showed that female TBI CON mice had higher perivascular AQP4 expression than female TBI SF-R mice. Whereas this deficit in perivascular AQP4 may be exacerbated in females, both sexes of TBI SF-R had the lowest mean expression of perivascular AQP4 (interaction effect Injury × SF-R; *F*_(1, 16)_ = 7.189, *p* < 0.05). There was a decrease in AQP4 polarization when comparing the proportion of perivascular to parenchymal expression at 20 μm from the vessel wall (main effect TBI; *F*_(1, 20)_ = 9.951, *p* < 0.01). *Post hoc* analysis showed that TBI SF-R had a significantly lower AQP4 polarization than sham SF-R; however, this TBI-induced deficit did not occur with TBI CON (Fig. 4D).

Because of the evidence of BBB disruption, we then labeled for the presence of peripheral immune cells with CD45 (Fig. 4E,F). TBI SF-R had the highest mean presence of CD45^+^ cells in the ipsilateral cortex per section (main effect injury; *F*_(1, 20)_ = 20.65, *p* < 0.01). *Post hoc* analysis showed TBI SF-R had significantly more CD45^+^ cells than sham SF-R. Altogether, these data show that TBI SF-R disrupts AQP4 expression and increases the presence of infiltrating cells in the ipsilateral cortex.

## Discussion

We have previously shown that chronic SF enhances inflammation and hippocampal-dependent deficits after TBI, but the lasting effects of post-injury SF were unknown. This study determined whether post-injury SF causes persistent behavioral and inflammatory deficits even with a period of recovery compared to TBI alone. We hypothesized that TBI would prevent recovery from SF relative to sham mice, resulting in persistent deficits compared to TBI CON. Here, we provide evidence that TBI limits recovery from SF as shown by divergent behavioral performance, glial reactivity, and BBB disruption.

One unique finding is the decrease in acquisition index of TBI SF-R mice compared to TBI CON. Overall, latency to reach the goal box was similar between experimental groups in the BMT, but TBI SF-R mice displayed unique within- and between-day performances. These results align with our previous work showing decreased acquisition of fear conditioning with post-TBI SF but no overall deficit of that fear memory.^43^ TBI, chronic stress, and sleep disruption have all been shown to cause or exacerbate spatial learning and memory deficits.^46,61,62^ Nonetheless, we did not detect a robust TBI effect in latency to reach the goal box. Although somewhat unexpected, these results are consistent with other publications examining the BMT in rats and mice several months post-injury^63^ and may reflect a variation in information processing between the TBI SF-R and TBI CON groups. Further work is needed to determine TBI and SF-induced changes in cognition and memory.

We found that TBI causes lasting gliosis, both dependent and independent of SF-R, in the cortex and hippocampus. TBI alone increased gliosis lateral to the lesion regardless of SF-R. Though independent of recovery after post-injury SF, this shows that lFPI causes long-lasting cortical glial reactivity. In the hippocampus TBI SF-R increased astrocytic reactivity in the ipsilateral CA1 that did not occur in TBI CON mice. This same region had exacerbated microglial reactivity associated with neuronal and behavioral deficits immediately after 30 days of post-TBI SF with no period of recovery. This persistent astrocytic reactivity mirrors the shift from microglial to astrocytic responses previously observed with a period of recovery after 3 days of acute sleep disruption post-TBI.^39^ Though subtle, these long-lasting glial responses can contribute to pathophysiology, cognitive deficits, and even neurodegeneration.^4,7,8,64,65^

Notably, there was a distinct neuronal signaling/maintenance signature with SF-R that was dependent on TBI. Inhibitory neurons consistent with the shared gene expression between SF-R groups play a major role in memory retrieval and could contribute to probe trial performance with SF-R.^66,67^ Many genes associated with neuronal maintenance and synaptogenesis were highly expressed with TBI SF-R compared to both TBI CON and sham CON. Whereas enhanced neuronal signaling has been observed acutely after TBI attributable to recovery from mechanical injury,^68,69^ chronic enhancement of these processes with post-injury SF-R could demonstrate maladaptive neuronal processing and contribute to cognitive deficits,^70^ particularly in GABAergic systems, post-TBI.^71^ Thus, dysfunctional maintenance of neuronal processing chronically after TBI and SF could contribute to a decreased acquisition index in the BMT, as well as provide pathways of interest for further investigation to better understand aberrant signaling that contributes to cognition after injury.

Finally, we found robust evidence of BBB disruption and infiltration of peripheral immune cells with TBI SF-R. The BBB is vulnerable to injury, given that both pre-clinical and clinical work has shown extensive BBB damage post-TBI.^72–74^ The BBB is also highly influenced by sleep^75^ and can be disrupted when sleep is altered. For example, 6 days of sleep restriction enhances BBB permeability.^76^ AQP4 regulates osmotic balance across the BBB and thus contributes to BBB maintenance and function.^77,78^ We previously reported that TBI and sleep disruption each decrease AQP4 polarization acutely after injury.^39,47^ Here, we show that AQP4 expression is differentially influenced by TBI with SF-R. AQP4 expression was enhanced with TBI alone whereas TBI SF-R had the lowest expression of perivascular AQP4. Further, this perivascular AQP4 expression had a sex-dependent response, with the TBI-induced enhancements of perivascular AQP4 being driven by males.

These studies were not powered to detect sex differences, but identification of sex-dependent responses builds on existing published data. For example, previous work similarly showed a sex-dependent difference in BBB permeability after controlled cortical impact experimental TBI, but there was no sex-dependent difference in infiltrating cells.^79^ This is notable given that we did not observe any sex-dependent changes in peripheral immune cell infiltration, but TBI SF-R had an increased presence of infiltrating immune cells compared to sham SF that did not occur with TBI CON. It is possible that increased AQP4 expression with TBI alone is compensatory and contributes to enhanced clearance of cells and debris, but this process is compromised by SF-R. Further work is needed to define how sex may influence BBB integrity chronically after injury and how that may influence the inflammatory post-injury environment. Additional studies are also needed to confirm how SF and SF-R may influence post-TBI BBB function and integrity.

In summary, TBI SF-R causes within-day spatial learning and memory deficits and enhanced expression of neuronal transcription and BBB dysfunction and increases the number of infiltrating immune cells. This work describes long-lasting responses to post-TBI SF that may not fully resolve. A variety of processes could contribute to these persistent dysfunctions, such as epigenetic changes attributable to both TBI and sleep restriction by SF.^80,81^ It is possible that further recovery from SF may eventually resolve post-TBI SF effects compared to TBI alone; however, repeated and varied stressors commonly occur throughout the life of TBI survivors. Persistent effects of post-TBI stress could thus compound with subsequent stressors. Altogether, these data emphasize the long-lasting effects that environmental factors have on TBI-induced neuroinflammation and function, even with a period of recovery.

## Abbreviations Used

ANOVA: analysis of variance
AQP4: aquaporin-4
BBB: blood–brain barrier
BMT: Barnes maze task
CON: control
DEGs: differentially expressed genes
DPI: days post-injury
GABA: gamma-aminobutyric acid
GFAP: glial fibrillary acidic protein
Iba-1: ionized calcium-binding adaptor molecule 1
lFPI: lateral fluid percussion injury
IPA: Ingenuity Pathway Analysis
SEM: standard error of the mean
SF: sleep fragmentation
SF-R: sleep fragmentation with recovery
TBI: traumatic brain injury
